# Generational perspectives on sustainable diets in Saudi Arabia: implications for food security and behavioral change

**DOI:** 10.3389/fnut.2025.1672606

**Published:** 2025-09-23

**Authors:** Sawsan Al-Hashim, Hala Hazam Al-Otaibi, Ahlam Saleh Alhajri

**Affiliations:** ^1^Department of Food and Nutrition Science, College of Agricultural and Food Science, King Faisal University, Al-Ahsa, Saudi Arabia; ^2^Department of Clinical Nutrition, College of Applied Medical Sciences, King Faisal University, Al-Ahsa, Saudi Arabia

**Keywords:** sustainable diets, food systems, plant-based protein, generational diets, nutritional transition, dietary behavior change

## Abstract

**Background and objectives:**

Sustainable healthy diets (SHDs) are pivotal for promoting public health while mitigating environmental impacts. However, the adoption of sustainable and healthy eating behaviors (SHEBs) varies across demographic groups, particularly generations. This study assessed generational differences in SHEBs, protein consumption patterns, motivations for dietary change, and readiness to adopt plant-based diets in Saudi Arabia, a nation undergoing rapid urbanization and dietary transitions.

**Methods:**

A cross-sectional online survey of 637 Saudi adults from Generations Z, Y, and X was conducted between November 2023 and March 2024. SHEBs were measured using a validated scale covering balanced diets, local food choices, meat reduction, food waste, and quality labels. Protein intake (animal- vs. plant-based), BMI, motivations for adopting SHDs, and stages of behavioral change were analyzed across generations using ANOVA, chi-square tests, and regression models.

**Results:**

Generation X exhibited the highest SHEBs scores, driven by quality-labeled food choices and reduced meat consumption. Generation Y showed moderate SHEBs engagement, motivated by ethical and environmental concerns. Generation Z reported the lowest SHEBs scores, with health and weight loss as primary motivators, but also the highest animal-protein intake. Across all generations, plant-based protein intake was a significant predictor of greater SHEBs adherence. However, over 80% of participants remained in the pre-contemplation or contemplation stages for adopting plant-based diets, highlighting behavioral resistance to change.

**Conclusion:**

Generational differences in dietary behaviors underscore the need for customized, generation-sensitive interventions to promote SHDs in Saudi Arabia. Enhancing awareness, addressing barriers to plant-based eating, and leveraging key motivators such as health and accessibility are essential steps toward transforming food systems for greater sustainability. These insights hold significant implications for developing generation-sensitive strategies that promote SHDs, improve nutritional outcomes, and strengthen food security in Saudi Arabia.

## 1 Introduction

Globally, urbanization has resulted in nutritional transitions that significantly contribute to the increasing prevalence of non-communicable diseases (NCDs), including obesity, cancer, type 2 diabetes, and cardiovascular diseases (1), which are accompanied by a higher intake of calories, trans fatty acids, saturated fatty acids, added sugars, sodium, and animal products (dairy and meat) (2). Simultaneously, factors such as increasing global population, climate change, high greenhouse gas emissions, decreased freshwater reserves, and biodiversity are serious threats to the environment, animals, and agricultural production (3, 4). Considering the strong association among global health, climate change, and food production, the global population needs to adopt safe, balanced, healthy, and adequate diets that are economically viable and affordable (5). Implementing these practices is particularly important for protecting the environment and health, preventing diseases, and promoting biodiversity by reducing environmental risk factors, and contribute to climate change mitigation (4, 6).

The term or concept of sustainable healthy diets (SHDs) was first introduced in 1986 (7). In 2019, FAO defined SHDs as, “*nutritionally adequate, safe and healthy diets that meet the nutritional needs of present and future generations, respect biodiversity and ecosystems, are protective, culturally acceptable, accessible, economically affordable, nutritionally adequate, safe and healthy*” (8). This concept has been widely adopted for all generations (9–11). A recent systematic review (5) found that adhering to SHDs containing predominantly plant-based calories and a majority (60%) of energy requirements could reduce mortality and mitigate the negative environmental impacts associated with diet.

SHDs are highly relevant today; however, it is crucial to determine whether all generations are familiar with this concept. Typically, individuals are exposed to similar historical and social contexts that are significantly influenced by their lifestyles and dietary behaviors across their lifespans, contributing to the development of different dietary habits and environmental concerns across generations. Generation X (GX) grew during a period of rapid modernization and adhered to traditional eating habits, with a strong commitment to the conventional nutritional norms. They focus on basic food groups and prefer foods from natural sources (12, 13). Generation Y (GY), shaped by the rapid technological growth of their time, smoothly adapted to these changes, embracing a fast-food culture characterized by quicker and more convenient eating habits. Despite this, GY also shows familiarity with healthy eating practices (14, 15). In contrast, Generation Z (GZ), who emerged in the era of advanced digitalization, are more likely to be concerned with environmental health and sustainable nutrition (16). Their dietary preferences are influenced by trends, including plant-based diets, organic product choices, and zero-waste movement. At the same time, fast-food culture, driven by the influences of social media and trends, continues to play a notable role in shaping their eating habits (17).

Dietary habits and environmental concerns differ significantly across generations. To promote SHDs practices that benefit both current and future generations, it is essential to assess the interconnectedness among human and environmental health.

Saudi Arabia (SA) is one of the fastest growing economies in the world that has reported a nutritional transition toward a modernized lifestyle, which is associated with a significant burden of NCDs and is responsible for 73% of all mortality (18–20). This is accompanied by a low intake of fruits and vegetables and an increased intake of animal products, refined foods, fast food, sodium, added sugars, saturated fatty acids, and trans-fatty acids (21). To address the significant burden of NCDs, the government of SA implemented the Healthy Food Strategy established by the Saudi Food and Drug Authority (22) as part of the Saudi Vision 2030 and aligned it with the Sustainable Development Goals (SDGs).

SA has undertaken significant efforts to achieve all SDGs related to sustainable environments (SDG 13, SDG 15), nutrition and food security (SDG 2, SDG 3), plant production and protection (SDG 2, SDG 15), natural resources (SDG 6, SDG 12), and waste management (SDG 12) (23), aligning them with the ambitious framework of Saudi Vision 2030 for all populations. Previous research conducted in SA has focused on sustainable environments and energy, climate change, and air quality practices (24, 25). To the best of our knowledge, very few studies have investigated SHDs and focused on one direction. Alnasser and Musallat (26) assessed food sustainability awareness to adopt sustainable food among SA citizens and reported lower awareness and understanding of the negative environmental impact of consuming unsustainable food. Another study investigated the understanding of SA citizens about climate change associated with dietary choices and reported poor understanding with a higher intake of non–climate-friendly foods (27). A nationwide study measured household food waste and reported a national prevalence of 63.6% for uncooked food waste and 74.4% for cooked food waste (28). However, the scenarios for plant-based food consumption vary. Recent research indicated a significant rise in the adoption of vegetarian and vegan diets (13%) among SA citizens, particularly among young adults (29, 30), accompanied by an increased awareness of the impact of dietary habits on health (31).

There is a research gap in SA where researchers aim to primarily focus on the insufficient comprehension of sustainable and healthy eating behaviors (SHEBs) among citizens. Previous studies have addressed food and environmental sustainability, food waste, knowledge of SHDs, and vegetarian and vegan diets (28–31). However, these efforts did not focus on the approaches of different generations (Z, Y, and X) in SA toward SHEBs and their readiness to modify or motivate their eating habits for more sustainable diets. The lack of comprehensive studies on generational differences in SA SHEBs including motivations, readiness for plant-based diets, and barriers to dietary change represents a critical gap. This is particularly significant given SA robust economic growth, the increasing prevalence of NCDs, and the government’s strong commitment to public health improvement, environmental sustainability, and achieving the SDGs. Therefore, this study aims to evaluate generational differences in SHEBs in Saudi Arabia, with a focus on protein consumption patterns across Generations Z, Y, and X. Additionally, the study investigates the key motivations and factors influencing the adoption of SHDs, including health, environmental, and cultural aspects. It further examines the readiness of different generations to adopt plant-based diets using the stages of behavioral change model.

## 2 Materials and methods

### 2.1 Sampling procedure

A convenience sample of Saudi adults (≥18 years) using a non-probability snowball sampling approach was recruited through WhatsApp-distributed Google Forms surveys between November 2023 and March 2024 (*N* = 637). Eligible participants were SA citizens who provided informed consent after reviewing study protocols. Participants were encouraged to forward the link to others different age or social circles groups to diversify the sample across generations. The sample size was determined *a priori* using G*Power 3.1. For generational comparisons (one-way ANOVA), we specified a small-to-medium effect size of *f* = 0.15, *α* = 0.05, power = 80%, and 3 groups (Gen Z, Y, X), yielding a minimum requirement of 159 participants (53 per group). Our final sample (*n* = 637) exceeded these thresholds, ensuring robust power even for subgroup analyses. The Scientific Research Ethics Committee of King Faisal University approved this study (KFU-REC-2023-JAN-ETHICS483) and was conducted according to the ethical principles expressed in the Declaration of Helsinki.

### 2.2 Study questionnaire

The questionnaire comprised six main sections, each adapted from previously developed questionnaires. A forward–backward translation was performed by bilingual experts to ensure cultural appropriateness. A pilot test was conducted with 20 Saudi adults from different age groups (GZ, GY, and GX) to evaluate the clarity, cultural appropriateness, and feasibility of the questionnaire. Minor revisions were made to improve wording and flow based on participants’ feedback. No significant structural changes were needed. The pilot participants reported that the questions were clear and easy to understand, and the average completion time was 10–15 min. These participants were excluded from the final analysis.

#### 2.2.1 Demographic data and anthropometric measurements

This section includes information on sex, age, educational level, monthly family income, and marital status. Participants were categorized into three groups based on their generation: “Generation X,” representing individuals born between 1966 and 1981 or aged 41–56 years; “Generation Y or Millennials,” representing those born between 1982 and 1995 or aged 26–40 years; and “Generation Z,” representing individuals born between 1996 and the present day or aged below 26 years (32). Height and weight were reported by the participants, and then the researcher calculated the BMI and classified it according to the WHO guidelines (1998) (33).

#### 2.2.2 Familiarity with a sustainable healthy diet

Participants’ familiarity with sustainable healthy diets was assessed through a single item measure Respondents were asked, “Are you familiar with the FAO definition of SHDs ‘*nutritionally adequate, safe and healthy diets that meet the nutritional needs of present and future generations, respect biodiversity and ecosystems, are protective, culturally acceptable, accessible, economically affordable, nutritionally adequate, safe and healthy*’ (8)?” with three response options: (1) Yes (familiar), (2) No (unfamiliar), and (3) I’ve heard the term but am unsure of its meaning (partial familiarity).

#### 2.2.3 Stage of change

Participants were asked to select one of six statements that best described their adherence to a plant-based diet. These statements were based on the six stages of change outlined in the Transtheoretical Model or Stage of Change (34). “I am not interested in following a plant-based diet at present or in the future” (pre-contemplation [PC] stage). “I am currently thinking about following a plant-based diet and may start within the next 6 months” (contemplation [C] stage). “I have decided to follow a plant-based diet in the near future” (preparation [P] stage). “I currently follow a plant-based diet” (action [A] stage). “I have been following a plant-based diet for more than 6 months.” (maintenance [M] stage). “In the past, I used to follow a plant-based diet, but I have now stopped.” (relapse [R] stage) (14). Some stages of change categories had a small number of responses, and the researchers combined them into three categories: PC and C, P and R, and A and M.

#### 2.2.4 Daily consumption of animal- and plant-based protein

The FFQ was adapted from Hu et al. (35) and modified to include food items and portion sizes commonly consumed in Saudi Arabia. However, the adapted FFQ was not formally validated against biomarkers or comprehensive dietary records in the Saudi population. To collect data on weekly consumption of both plant and animal proteins by the participants, such as red meat, chicken, dairy, eggs, fish, and dairy products. Plant-based proteins included nuts, legumes, processed meat substitutes, and plant-based milk. The participants were asked to indicate their frequency of consumption by choosing from a range of options, such as “four or more per day” (equivalent to 28 portions/week), “three per day” (equivalent to 21 portions/week), “two per day” (equivalent to 14 portions/week), “one per day” (equivalent to seven portions/week), “five to six per week” (equivalent to 5.5 portions/week), “two to four per week” (equivalent to three portions/week), and “one per week” (equivalent to one portion/week) (36). The upper limit was set at 35 portions/week, which is equivalent to five portions/day. This upper limit was chosen to ensure that responses from the participants remained within a reasonable range, thus preventing extreme values from skewing the data. The total protein consumption was calculated by summing the weekly portions of all protein items (separately for animal, plant-based protein). The portion size and amount of protein were determined according to Hagmann et al. (36) and Żakowska-Biemans et al. (37).

#### 2.2.5 Sustainable and healthy eating behaviors

Żakowska-Biemans et al. (37) developed and validated Sustainable and Healthy Eating Behaviors (SHEBs), which is widely used (38, 39) for evaluating the relationship among human, animal, and environmental health for fostering long-term sustainability. It encompasses eight components with a 34-item scale addressing diverse aspects, including: “Healthy and balanced diet” (10 items), “Regional and organic quality labels” (five items), “Reducing meat consumption” (four items), “Local foods” (three items), “Low-fat products” (three items), “Food waste” (three items), “Animal welfare” (three items), and “Seasonal food” (three items). Participants were asked to rate their levels of engagement in these eating behaviors on 7-point Likert scale ranging from “Never = 1” to “Always = 7” (37). The scale scores were calculated by averaging the scores assigned to the items in each component. To calculate the total components, score, the average of the scores for all dimensions was collected. Higher average scores were associated with a higher number of SHEBs. Cronbach’s alpha coefficients ranged from 0.904 to 0.908 for the dimensions and 0.909 for the total dimension, which was comparable with the previous studies (38, 39). The Arabic version was developed through forward–backward translation and piloted among 20 Saudi adults.

#### 2.2.6 Motives to choose sustainable healthy diet

Participants identified their primary motivations for adopting SHDs by selecting from nine predefined options: health, accessibility, taste preferences, religious considerations, environmental sustainability, animal welfare, weight management, cost, and sensory appeal. Multiple selections were permitted to capture the complexity of dietary decision-making (14, 40).

### 2.3 Statistical analysis

Data was analyzed using IBM SPSS Statistics (v.29) after assessing normality through Shapiro–Wilk tests, Q-Q plots, and histograms. Descriptive statistics (means ± SDs, frequencies) characterized the sample, while one-way ANOVA test was performed for continuous variables, and chi-square analysis was used for categorical variables. Motivation for adopting SHDs was analyzed using multiple response analysis. To compare generational differences in the proportion selecting each motivation, a series of chi-square tests were conducted, treating each motivation as a binary variable (selected vs. not selected). Linear regression modeled SHEBs against BMI, protein intake, and socioeconomic factors across generations. Only the variables with significant differences were included in the model, statistical significance was set at *p* < 0.05.

## 3 Results

A total of 637 participants completed the survey, the majority were from GZ and GY, with significant differences between the generations (*p* < 0.000); more participants were educated to university or higher levels, married (except GZ), and had higher income with significant differences (*p* = 0.003, *p* = 0.000, and *p* = 0.04, respectively) (Table 1). The participants were also questioned about their familiarity with SHDs. Of all, 43% of the participants reported that they were unfamiliar with the concept, whereas approximately 33% of those partially familiar they had heard about it but lacked a clear understanding. Only 24% of the participants are familiar with SHDs, representing a minority of the overall sample. Notably, the results indicated that GY reported greater familiar with SHDs than the other generations, without a significant difference (Table 1).

**Table 1:** Participant demographics and familiarity with a sustainable healthy diet by generation (*n* = 637).

Variable	All participants	Generation Z (< 26) 398 (62.48%)	Generation Y (26–40) 141 (22.14%)	Generation X (41–56) 98 (15.38%)	*P*-value
Mean ± (SD) or *n* (%)					
Age	27.84 ± 10.529	21.22 ± 2.117	32.23 ± 3.794	48.46 ± 7.084	0.000^***^^a^
Sex					
Male	194 (30.45)	103 (25.87)	44 (31.20)	47 (47.95)	0.000^***^^a^
Female	443 (69.54)	295 (74.12)	97 (68.79)	51 (52.04)	
Marital status					
Married	264 (41.44)	116 (29.14)	88 (62.41)	60 (61.22)	0.000^***^^b^
Single	373 (58.55)	282 (70.85)	53 (37.58)	38 (38.77)	
Educational level					
Secondary school or lower	174 (27.31)	124 (31.15)	23 (16.31)	27 (27.55)	0.003^**^^b^
University or higher	463 (72.68)	274 (68.84)	118 (83.68)	71 (72.44)	
Monthly family income					
SAR 10,000 or less	225 (35.3)	144 (36.18)	53 (37.58)	28 (28.5)	0.04*^b^
More than SAR 10,000	412 (64.7)	254 (63.81)	88 (62.41)	70 (71.5)	
Familiarity with a sustainable healthy diet					
Familiar	153 (24.01)	94 (23.61)	36 (25.53)	23 (23.46)	0.850^b^
Unfamiliar	274 (43.01)	172 (43.21)	56 (39.71)	46 (46.93)	
Partial familiarity	210 (32.96)	132 (33.16)	49 (34.75)	29 (29.59)	

a ANOVA test.

b Chi-square test.

* *p* < 0.05; *** *p* < 0.0001. SD, standard deviation.

Table 2 presents anthropometric measurements, weekly protein consumption, and stages of change. GY and GZ reported mean body mass index (BMI) values within the normal category (23.9 ± 5.35 and 24.31 ± 6.51, respectively), whereas GX had BMI in the overweight category (26.47 ± 6.03), with a significant difference between generations (*p* = 0.000). GZ reported a lower body weight (60.24 ± 17.95) than GY and GX (*p* = 0.000). Notably, 47.6% of the total samples were in the normal weight category, whereas 13.2% were underweight. Additionally, a higher proportion of GX were overweight and obese and had the highest weight (75.06 + 15.14) compared to the others, with a significant difference (*p* = 0.001 and *p* = 0.000, respectively). GZ reported consuming higher mean daily portions of animal-based protein (9.67 ± 4.74), and GX reported consuming higher means (5.50 ± 4.14) daily portions of plant-based protein, with a significant difference (*p* = 0.044 and *p* = 0.03, respectively). Most participants (82.57%) were either uninterested in or only thought (PC/C stages of change) about the adoption of a plant-based diet. Only 4.39% of the participants were already in A/M stages of change for adopting a plant-based diet, without significant difference between the generations.

**Table 2:** Generational differences in BMI, protein consumption, and readiness for plant- based diets (*n* = 637).

Variable	All participants	Generation Z (<26) 398 (62.48%)	Generation Y (26–40) 141 (22.14%)	Generation X (41–56) 98 (15.38%)	*P*-value
Mean ± (SD) or *n* (%)					
Weight (kg)	64.35 ± 17.72	60.24 ± 17.95	66.41 ± 15.4	75.06 + 15.14	0.000^***^^a^
BMI (kg/m^2^)	24.41 ± 6.26	24.31 ± 6.51	23.29 ± 5.35	26.47 ± 6.03	0.000^***^^a^
BMI categories					
Underweight	84 (13.18)	59 (14.78)	24 (17.02)	1 (1.02)	0.001^**^^b^
Normal weight	303 (47.56)	187 (46.98)	73 (51.77)	43 (43.87)	
Overweight	170 (26.68)	103 (25.87)	29 (20.56)	38 (38.77)	
Obese	80 (12.55)	49 (12.31)	15 (10.63)	16 (16.32)	
Daily portions consumption					
Animal-based protein	9.55 ± 4.46	9.67 ± 4.74	9.36 ± 3.71	8.11 ± 4.22	0.044^*^^a^
Plant-based protein	4.86 ± 4.19	4.91 ± 4.42	4.46 ± 3.45	5.50 ± 4.14	0.031^*^^a^
Stages of change					
PC/C	526 (82.57)	331 (83.16)	118 (83.68)	77 (78.57)	0.646^b^
P/R	83 (13.02)	52 (13.06)	17 (12.05)	14 (14.28)	
A/M	28 (4.39)	15 (3.76)	6 (4.25)	7 (7.14)	

a ANOVA test.

b Chi-square test.

* *p* < 0.05; ** *p* < 0.001; *** *p* < 0.0001. SD, standard deviation; BMI, body mass index; PC, pre-contemplation; C, contemplation; P, planning; R, relapse; A, action; M, maintenance.

The total and dimension scores on the SHEBs scale for GZ, GY, and GX participants are shown in Table 3. The total score on the SHEBs scale of the participants from all generations was significantly different (*p* < 0.007), with higher a mean for GX (3.18 ± 0.43). The healthy and balanced diet dimension mean score was higher in GX (7.10 ± 1.62) than GY (6.04 ± 1.47), whereas it was significantly (*p* = 0.001) lower in GZ (5.62 ± 1.48). GX were more concerned about quality labels (2.98 ± 0.88) and meat reduction (1.98 ± 0.60) (*p* = 0.011 and *p* = 0.003, respectively). GZ focused more on buying and eating local foods (0.91 ± 0.48, *p* = 0.025). These dimensions were not significantly different between generations low fat, food waste, animal welfare, and seasonal foods.

**Table 3:** Dimensions of sustainable and healthy eating behaviors (*n* = 637).

Variable	All participants	Generation Z (<26) 398 (62.48%)	Generation Y (26–40) 141 (22.14%)	Generation X (41–56) 98 (15.38%)	*P*-value
Mean ± (SD)					
Healthy balanced diet	5.79 ± 1.52	5.62 ± 1.48	6.04 ± 1.47	7.10 ± 1.62	0.001^**^
Quality labels	2.23 ± 0.91	2.1 ± 0 0.88	2.16 ± 0.95	2.98 ± 0.88	0.011^*^
Meat reduction	1.70 ± 0 0.62	1.64 ± 0 0.62	1.73 ± 0.60	1.98 ± 0 0.60	0.003^**^
Local foods	0.78 ± 0 0.48	0.91 ± 0.48	0.71 ± 0.47	0.80 ± 0.46	0.025^*^
Low fat	1.13 ± 0.57	1.10 ± 0 0.57	1.16 ± 0.56	1.21 ± 0.53	0.190
Food waste	1.76 ± 0 0.37	1.73 ± 0.40	1.78 ± 0.32	1.81 ± 0.30	0.141
Animal welfare	1.09 ± 0 0.47	1.12 ± 0 0.47	1.02 ± 0 0.45	1.10 ± 0 0.45	0.105
Seasonal food	0.96 ± 0.45	0.95 ± 0 0.43	0.99 ± 0.49	0.95 ± 0.43	0.681
Total SHEBs	2.07 ± 0.43	2.03 ± 0.42	2.08 ± 0 0.43	3.18 ± 0.43	0.007^**^

^a^ ANOVA test; * *p* < 0.05; ** *p* < 0.001. SD, standard deviation.

Table 4 presents motivations for choosing SHDs by generation, the top response was health (91.99%), followed by weight loss (49.60%) and enjoyment (37.99%). The lowest response was for animal welfare (6.12%), followed by environmental sustainability (17.73%); however, GY reported a slightly higher motivation to SHDs (21.98%) than GZ and GX. Accessibility was an important dimension for 24.48% of GX. Taste was the only motivator with a statistically significant difference between the generations (*p* = 0.047). Generation X reported higher taste motivation (39.79%) than Gen Z (28.39%) and Gen Y (32.62%). For all other motivations, there were no significant generational differences.

**Table 4:** Motivations for choosing sustainable healthy diets by generation (*n* = 637)^*^.

Variable	All participants	Generation Z (<26) 398 (62.48%)	Generation Y (26–40) 141 (22.14%)	Generation X (41–56) 98 (15.38%)	*P*-value
*n* (%)					
Health	586 (91.99)	368 (92.46)	130 (92.19)	88 (89.79)	0.122
Price	101 (15.85)	57 (14.32)	28 (19.85)	16 (16.32)	0.084
Taste	198 (31.08)	113 (28.39)	46 (32.62)	39 (39.79)	0.047*
Animal welfare	39 (6.12)	19 (4.77)	13 (9.21)	7 (7.14)	0.432
Accessibility availability	120 (18.83)	74 (18.59)	22 (15.60)	24 (24.48)	0.112
Religious	117 (18.36)	70 (17.58)	29 (20.56)	18 (18.36)	0.891
Environmental sustainability	113 (17.73)	65 (16.33)	31 (21.98)	17 (17.34)	0.431
Weight loss	316 (49.60)	197 (49.49)	76 (53.90)	43 (43.87)	0.056
Enjoy	242 (37.99)	148 (37.18)	55 (39)	39 (39.79)	0.614

* Participants could choose more than one dimension; * *p* < 0.05.

Table 5 presents the results of linear regression analysis conducted individually for each generation to identify the predictors of SHEBs. The results indicated that consuming portions of plant-based protein daily was a predictor of more SHEBs among GZ (*β* = 0.177, *p* = 0.002) and GY (*β* = 0.344, *p* = 0.000). Higher BMI (*β* = 0.223, *p* = 0.028), consuming portions of plant-based protein (*β* = 0.235, *p* = 0.026) on a regular basis, and consuming lower portions of animal-based protein (*β* = −0.217, *p* = 0.032) daily, were predictors of more SHEBs among GX. Finally, lower body weight (*β* = 0.125, *p* = 0.013) was predicted for more SHEBs among GZ.

**Table 5:** Generational predictors of sustainable healthy eating behaviors (*n* = 637).

Generation groups	Variable	*β*	SE	*P*-value
Generation Z (<26)	Sex	0.028	0.049	0.578
	Marital status	−0.008	0.046	0.867
	Educational level	−0.006	0.046	0.898
	Income	−0.053	0.044	0.299
	Weight	0.125	0.001	0.013*
	BMI	−0.021	0.003	0.672
	Animal-based protein	0.045	0.005	0.377
	Plant-based protein	0.177	0.005	0.002^*^
Generation Y (26–40)	Sex	0.202	0.087	0.331
	Marital status	−0.05	0.073	0.535
	Educational level	0.008	0.099	0.923
	Income	0.033	0.077	0.701
	Weight	−0.12	0.002	0.155
	BMI	−0.064	0.007	0.437
	Animal-based protein	−0.01	0.01	0.908
	Plant-based protein	0.344	0.011	0.000^***^
Generation X (41–56)	Sex	0.081	0.094	0.459
	Marital status	0.047	0.089	0.646
	Educational level	0.048	0.1	0.648
	Income	−0.172	0.093	0.116
	Weight	−0.097	0.003	0.344
	BMI	0.223	0.007	0.028^*^
	Animal-based protein	−0.217	0.011	0.032^*^
	Plant-based protein	0.235	0.011	0.026^*^

* *p* ≤ 0.05; *** *p* ≤ 0.0001; BMI, body mass index.

## 4 Discussion

In recent years, the growing focus on sustainability has been motivated by the pressing concerns presented by climate change. For this purpose, in 2015, the UN established the SDGs, aiming to “peace and prosperity for people and the planet” which prioritized SHDs that could provide all essential nutrients in quantities tailored to the needs of an individual based on their bodily requirements for present and future generations, with respect to ecosystems, biodiversity, and minimizing harm to the environment. Cultural acceptability, wide accessibility, and economical feasibility were considered in this process of prioritizing SDGs. This study was conducted to assess the association between SHEBs and other potential factors, including socioeconomic status, motivation, anthropometric measurements, and readiness to adopt a plant-based diet in three different generational groups GZ, GY, and GX in SA.

Female participants were overrepresented because women are more interested in nutritional and health-related issues than men and are more willing to adopt plant-based diets and seasonal products (41). However, previous evidence indicates that younger individuals are more likely to adopt SHEBs, such as organic and local foods (42). A majority of GY and GX were married and had higher monthly incomes, which is understandable given that individuals aged more than 26 years are typically in relationships and are economically stable. Most GZ, unmarried where single individuals often eat less healthily due to a lack of motivation to cook for one, and the absence of social support and accountability from others can make it harder to maintain good dietary habits.

The familiarity with SHDs in SA has not reached the optimal level, and only a few studies have been conducted on familiarity with SHDs. Alnasser and Musallat (26) assessed awareness among SA citizens about sustainable food practices and reported that the awareness was low, with a limited understanding of how consuming unsustainable food impacts the environment negatively. Similarly, another study examined understanding of climate change among SA citizens in relation to dietary choices and found limited familiarity and higher consumption of non–climate-friendly foods (27). The findings of the present study were comparable with those of previous studies conducted in SA, revealing that not all generations were familiar with SHDs, or that they lacked a clear understanding of their meaning. Approximately one-fifth of the participants were familiar with SHDs.

Twenty-five percent of GY were familiar with SHDs, with 83.68% having attained a higher level of education, which aligns with the findings of Culliford and Bradbury (14) who reported that individuals aged 35 years and older, particularly highly educated women, were more familiar with the environmental benefits of adopting sustainable food practices. In contrast, Acar Tek et al. (43) reported limited familiarity levels, with only 26.6% of GZ, 20.3% of GY, and 16.6 of GX having heard of a sustainable diet, which is lower than those reported by the present study. Furthermore, several other studies have revealed that a small proportion of adults possess a good understanding of sustainable diets (44).

Most participants across all generations were within the normal-weight category, and the highest percentage of participants with normal weight was in GY. However, 39% of GX are overweight and 16% are obese, possibly because of their inactive lifestyles and slower metabolism associated with fat accumulation in the body (45). In this context, the findings of this study differ from those of Martinson et al. (46), who reported that obesity is significantly more prevalent among GY than among GX in the United States, especially among men. The study also found that GY women in England were more likely to be obese than GX women, whereas in the United States, the disparity in obesity rates between the two generations of women was not significant. The differences between the present study and Martinson et al. (46) could be due to regional differences in diet, physical activity, socioeconomic status, and healthcare access, which contribute to contrasting rates of obesity.

Consumption of animal-based proteins is the most energy-intensive and environmentally significant concern, especially in the livestock sector. Plant-based proteins can lower greenhouse gas emissions and provide an important strategy for environmental sustainability (47). In SA, urbanization and rising income are key factors in the westernization of dietary habits. This shift is characterized by an increased consumption of animal-based products, energy-dense foods, and ultra-processed foods. Animal-based protein consumption showed that GZ had the highest weekly protein consumption, whereas GX had the lowest. Conversely, the GX group showed the highest weekly consumption of plant-based proteins. Notably, GY ranked second in consumption of both animal- and plant-based proteins. Traditionally, the consumption of animal-based proteins, especially red meat, in SA is influenced by various variables, including cultural and social elements, and serving generous portions of meat is regarded as a symbol of hospitality and generosity on all occasions. The average daily consumption of red meat by SA citizens is 73.26 g per day (48). Another explanation that could support this behavior is the genuine lack of information about the environmental benefits of plant-based protein consumption such as the lowering of carbon emissions.

A cross-cultural study by Migliavada et al. (32) aimed to examine how impulsive traits and individuals’ knowledge of sustainable food impact the frequency of animal- and plant-based food consumption. Among participants in Turkey, the consumption of animal- based food was significantly (*p* = 0.03) higher for GY (*p* = 0.03), but among those in Italy, GZ showed a significantly (*p* = 0.04) higher consumption of animal-based foods compared with GY. Regarding consumption of plant-based foods, no significant generational differences were reported between the Italian and Turkish populations. However, participants in Italy showed a significantly higher consumption of plant-based foods.

Ruzgys and Pickering (49) found that GZ were reluctant to decrease meat consumption because of a disconnect between their beliefs and actions regarding sustainable diets. The motivation for adopting certain sustainable practices may stem more from health considerations than from environmental benefits. Only 55% of the GZ participants perceived a reduction in red meat consumption as beneficial to the environment, but few were willing to reduce meat consumption for environmental reasons.

The results revealed that most participants unwilling to adopt a plant-based diet across the three generations were in the PC/C stage, with a relatively few in A/M and P/R stages. Notably, most participants from GZ and GY (83.00%) were in the PC/C stages, whereas GX had a higher proportion of participants in A/M and P/R stages (21.24%) than GZ and GY (16.00%). This observation could be due to the unwillingness of participants to fully adopt plant-based diets, as there are some potential obstacles and facilitators for consumption, such as insufficient information, difficulty in developing new cooking skills, and positive expectations for the flavor of plant-based diets (50). According to a study (51), GZ has strong positive attitudes toward environmental concerns; however, if these concerns do not directly impact their lives, it may be because of their hesitation to adopt to plant-based diets owing to the difficulty of making a complete switch.

Health was the first motivator across all generations to adopt SHDs, as shown in previous studies (14, 26, 30, 52). However, dietary guidelines across the world focus only on health without environmental considerations, as observed in the present study, and environmental sustainability is ranked as the second-to-last motivator before animal welfare across all generations. For many people, environment related concerns are increasing, but may not be as central to decision making as personal health. Similarly, animal welfare is important, but is viewed as a secondary issue, which contributes to its lower ranking compared with other motivators. The second motivator was weight loss, especially in GY (53.9%), of whom more than half of the participants were categorized as having a normal weight. This may be because individuals in this age group are more health conscious and aware of the long-term benefits of maintaining a healthy weight compared with other generations (14, 32).

Fostering long-term sustainability requires measuring interdependence among the environment, animals, and human health. Participants who scored the highest on the dimension of healthy and balanced diet, food waste, and quality labels showed the most potential to adopt SHEBs, which is in line with the results from other studies evaluating SHEBs using the same scale (39, 53, 54). The total score of GX participants on the SHEBs scale was significantly higher (*p* = 0.007) than that of GZ and GY participants. This finding suggests that the GX participants may demonstrate a greater willingness to adopt SHEBs.

Linear regression analysis revealed the key predictors of SHEBs across generations, with variations in their influencing dimensions. Higher body weight and increased consumption of plant-based proteins were predictors of more SHEBs adoption in GZ. Interestingly, this result contrasts with the findings of a previous study (53), which showed that lower SHEBs scores were associated with obesity. This observation suggests that for the GZ, adopting plant-based diets may offset the negative association between higher body weight and healthy eating behaviors. Forty-three percent of GZ are unfamiliar with SHDs, with 83% in PC/C stages demonstrating a lack of interest in change and adoption of SHDs. Usually, SHDs are expensive, which is a major barrier to adopting them. Young adults in Poland opined that SHDs were closely associated with health and balance, but they were less available and more expensive than other diets (37). GZ are typically not committed to adopting SHDs, as many of them are university students who live away from home, which significantly influences their eating habits.

The consumption of plant-based proteins in GY was a significant predictor of higher adoption of SHEBs. These findings align with the results of other studies, such as those of Migliavada et al. (32), highlighting the positive relationship between plant-based protein intake and SHDs in GY. They exhibit traits from both generations, older (GX) and the youngest (GZ), most of whom are married; have a higher level of education; only 10.63% are obese, with more than 50.00% having a normal weight; and almost 26.00% are familiar with the meaning of SHDs. GY ranked second after GX in A/M stages and had the same percentage in PC/C stages (83.00%) as GZ in adopting SHDs. A possible reason for the presence of most participants in the PC/C stages is that young GY typically practice different eating behaviors than older generations (55). Notably, almost 40% of GY, like GZ, have a low income, which may affect their ability to adopt SHDs, resulting in the selection of fewer SHD options aligned with their financial limitations. Environmental sustainability (22.00%), religious beliefs (21.00%), and animal welfare (9.00%) were the key motivations driving GY to adopt SHDs more than GZ and GX. This may be attributable to GY’s greater knowledge of SHDs related to environmental and ethical issues together with a strong inclination toward aligning their dietary choices involving personal values and social responsibilities. Gala et al. (55) and DePew and Gonzales (56) reported different findings among adults in the United States where GY were more likely to be obese than GX, had low personal values and social responsibility, and reported poor self-esteem, followed by GX.

GX reduced the consumption of animal-based proteins, increased the intake of plant-based proteins, and had higher BMI as a predictor of more SHEBs. This result indicates that GX participants may have been more conscious of balancing their dietary choices with sustainable practices, possibly influenced by a higher percentage (55.00%) of overweight or obese participants. However, most GX participants were willing to adopt SHDs compared with other generations (21.24% in stages P/R and A/M). Our findings are consistent with previous studies, which found that older adults are more receptive to adopting SHDs than younger ones (14, 32, 39). The most important dimensions affecting GX were the adoption of SHEBs, quality labels, reduction in meat consumption, and food waste. Swiss participants also reported a positive perception on local and seasonal foods and that placing organic brands often reduces environmental impact (57). Notably, more than 60% of GX are married, which may influence their beliefs and behaviors, and have more SHEBs, making them the most willing generation to adopt SHDs compared with other generations. Previous studies suggest that married individuals tend to prioritize their health, both for the well-being of their families and because of the stability in relationship that marriage often provides (14, 32).

This study has several strengths. First, to the best of our knowledge, this is the first study to evaluate the perspectives on adoption of SHEBs among adults in SA across generations and fill a significant research gap. Second, this study offers valuable information on dietary behaviors and motivations across different generations. Third, the study investigates multiple factors, including socioeconomic status, protein consumption, readiness to adopt plant-based diets, anthropometric measurements, and motivational variables, to provide a comprehensive understanding of these factors. Fourth, the study used validated and reliable tools to assess participants’ SHEBs and ensured accuracy during data collection. Finally, the large sample size involving 637 participants from different generations provided cultural and environmental variation and good statistical power.

The present study has several limitations, the first of which is its cross-sectional design, which makes it difficult to determine the course and effect, and we cannot capture changes in behaviors over time. Second, the FFQ was culturally adapted and pilot tested, it was not fully validated for the Saudi population using reference dietary assessment methods. This could affect the precision of reported protein intake estimates. Third, dietary behaviors and anthropometric measurements of self-recall can cause over or underestimation owing to social desirability. Fourth, despite the efforts to reduce bias through anonymous responses and broad recruitment, the use of an online convenience sampling approach may have introduced selection bias, with possible underrepresentation of individuals who are less active online or less comfortable with digital tools. Response bias cannot be fully excluded due to the self-reported nature of the data. Fifth, the one-item measure familiarity with SHDs does not reflect the level of interest surrounding the adoption of SHDs, which can change over time owing to external factors. Sixth, almost 70% of the participants were women who were more inclined to complete questionnaires related to health to express their opinions, unlike men who may quit early. Finally, our sample was not representative of the general population in SA.

## 5 Conclusion

This study reveals critical generational gaps in the understanding and adoption of SHDs in Saudi Arabia. While health remains the strongest motivator, environmental sustainability and animal welfare are secondary considerations, particularly among younger generations. The findings emphasize the urgent need for tailored educational and policy interventions that address specific generational needs and barriers. Encouraging plant-based protein consumption, reducing food waste, and promoting local food sources can collectively support health and environmental goals. SA commitment to Vision 2030 offers a strategic framework to foster these dietary shifts, contributing to climate change mitigation and food security.

## Data Availability

The raw data supporting the conclusions of this article will be made available by the authors, without undue reservation.
